# Advances in Analytical Strategies to Study Cultural Heritage Samples

**DOI:** 10.3390/molecules28176423

**Published:** 2023-09-04

**Authors:** Maria Luisa Astolfi

**Affiliations:** 1 Department of Chemistry, Sapienza University of Rome, Piazzale Aldo Moro 5, 00185 Rome, Italy; marialuisa.astolfi@uniroma1.it; 2 Research Center for Applied Sciences to the Safeguard of Environment and Cultural Heritage (CIABC), Sapienza University of Rome, Piazzale Aldo Moro 5, 00185 Rome, Italy

The advancements of civilization are based on our ability to pass on the events and knowledge of the past so that the next generations can start from an ever-higher level of expertise. The memory of the past can be kept alive by preserving historical and artistic assets, the cultural heritage of every nation.

In light of the above, this Special Issue (S.I.) aimed to collect studies that describe interesting/relevant problems in analyzing cultural heritage samples and suggest analytical strategies to solve/manage them. A total of 19 manuscripts were published (16 original research articles and 3 reviews). The submitted papers cover different aspects of cultural and archeological heritage protection, conservation, and restoration.

The sample matrix complexity belonging to cultural heritage, including archeological samples, generally requires a multi-analytical approach. The integrated use of different analytical techniques, with the preference for noninvasive or micro-invasive ones, allows an in-depth understanding of the original materials and their degradation processes and helps obtain innovative solutions for the restoration and conservation of artworks.

The paper by Cotte et al. [1] shows the advantages of techniques based on synchrotron radiation and X-ray powder diffraction (XRPD) to study ancient cultural heritage materials. In particular, high-angular-resolution X-ray powder diffraction (HR-XRPD) allows accurate characterization of crystalline materials, while micro X-ray powder diffraction (µXRPD) mapping provides additional information on the 2D or 3D distribution of crystalline phases at the micrometer scale. The authors outline the potential of these instrumental techniques, specific hardware and software developments to facilitate and speed up data acquisition and processing, and show recent applications to pigments, paints, ceramics, and wood.

Recently, enormous advances have been seen in analytical strategies for studying bioarcheological materials, particularly through mass spectrometry. The work of Barberis et al. [2] offers a focused approach to the chemical characterization of human tissues, embalming compounds, and layering organic ceramic materials. The authors applied a rapid and noninvasive functionalized film method to collect various compounds, ranging from macromolecules such as proteins to small molecules such as organic acids, and perform nontargeted analysis of the human remains of an Egyptian 18th dynasty individual named Nebiri.

The synergistic action of air pollution, climatic conditions, and biological contamination can harm the preservation of historical and artistic heritage. In particular, prehistoric artworks, including paintings and engravings, are particularly fragile as they are exposed to different environmental impacts. With the use of nondestructive elemental and molecular spectroscopies, it is possible to choose the most appropriate conservation and restoration strategy for prehistoric artworks and better understand their evolution over time.

In work from Costantini et al. [3], micro-energy dispersive X-ray fluorescence spectroscopy (µ-EDXRF), Raman spectroscopy, and X-ray diffraction (XRD) enabled the characterization of the degradation product, mineral substrate, and pigments in microsamples belonging to the open-air rock art site of Cueva de la Vieja (Alpera, Albacete, Spain). In addition, an extensive phenomenon of biological activity was also found in almost all samples analyzed.

The degradation of stone materials with the formation of unsightly and chromatic stains may be related to the biological activity of specific microorganisms and promoted by environmental factors such as temperature, humidity, and illumination. The work by Cardellicchio et al. [4] investigates the degradation on a hypogeum wall of the “San Pietro Barisano” rupestrian church, located in the Sassi of Matera (Italy), one of the UNESCO World Heritage Sites. The authors present an innovative and ecological approach using the biocidal action of a mixture of natural glycoalkaloids extracted from the unripe fruit of Solanum nigrum. The disappearance of the biological patina was assessed visually and using X-ray photoelectron spectroscopy (XPS), scanning electron microscopy (SEM), and energy dispersive X-ray spectroscopy (EDS). Bio-patina analyses revealed the bio-calcogenicity of some native microorganisms, which could, once the bio-cleaning phase was completed, be isolated and reintroduced on the wall surface to act as consolidants.

Among the various forms of degradation affecting stone artifacts, colored stains that form on surfaces in contact with metals or alloys are of particular concern. In the work of Reale et al. [5], the mechanisms of rust formation and diffusion in carbonates are defined. The different oxidation states of Fe are studied through the combined use of various analytical techniques, such as optical microscopy (O.M.), SEM-EDS, XPS, and Mössbauer spectroscopy. A better understanding of the composition and evolution of iron-based stains may enable more proper care of archeological artifacts and specific treatments for more effective and safe rust removal.

Studies of Fe speciation in archeological ceramics may be critical to gain insights into the various types of clay used and the different techniques and temperatures of clay firing. The study by Kozak et al. [6] reports the results of Fe speciation in ceramics obtained from archeological sites. UV-Vis molecular absorption spectrophotometry was used to determine Fe(II) and Fe(III). At the same time, the elemental composition was determined by inductively coupled plasma optical emission spectrometry (ICP-OES) and EDXRF spectrometry.

Among the most beautiful expressions of ancient art is the Roman mosaic. The preliminary study by Colantonio et al. [7] suggests a multi-analytical approach using SEM-EDS, XRF, nuclear magnetic resonance (NMR), Fourier-transform infrared spectroscopy (FT-IR), and gas chromatography–mass spectrometry (GC-MS) to assess the causes of chemical degradation of a black-and-white Roman mosaic at Palazzo Valentini near the Roman Forum (Italy). The study’s results may improve the knowledge of the Roman mosaic and its construction phases and enable the most appropriate strategies for its conservation to be undertaken.

Another topic addressed in this S.I. is the study and evaluation of the authenticity of precious stones belonging to cultural heritage. The work of Aceto et al. [8] suggests the use of UV-visible diffuse reflectance spectrophotometry with fiber optics (FORS) as a rapid and simple method for the preliminary identification of gemstones. It describes applying chemometric pattern recognition methods for processing large spectral datasets.

Two papers, by Serafini et al. [9] and Bosi et al. [10], focus on the study of dyes. In the former, the authors present and successfully apply an ammonia-based extraction protocol and an innovative purification step based on the use of an ion-pair dispersive liquid-liquid microextraction (IP-dLLME) for the purification and preconcentration of synthetic dyes before high-performance liquid chromatography (HPLC-HRMS) analysis. Synthetic dyes represent a relatively new field in cultural heritage analysis and are becoming increasingly important due to the growing demand for conservation interventions in 19th–20th century art productions. The case study of Serafini et al. [9] illustrates the advantages and need for a multi-analytical approach to analyzing unknown artifacts. The combined use of Raman spectroscopy and mass spectrometry can help interpret spectra and determine compounds in synthetically dyed objects [9].

The work from Bosi et al. [10] highlights the importance of developing analytical methods to preserve integrity and obtain increasingly comprehensive information on unique objects with priceless historical or artistic significance. The authors propose a minimally invasive approach for identifying madder, turmeric, and indigo dyes via hydrogel-supported extraction and subsequent surface-enhanced Raman spectroscopy (SERS) analysis on the gel after the addition of colloidal Ag pastes. In situ gel-supported microextraction was suitable for multi-analytical dye identification in wool and tempera by SERS and HPLC-MS/MS, after appropriate re-extraction and purification of the analytes [10].

Noninvasive cross-sectional analysis of pictorial stratigraphy in paintings, essential for adopting the most appropriate cleaning strategies or assessing adhesion and compactness between layers, is a major challenge in cultural heritage science. To date, no technique can noninvasively and uniquely measure the thickness of pictorial layers, as these are generally composed of heterogeneous and optically opaque materials. In the work of Dal Fovo et al. [11], the possibility of obtaining stratigraphic information on the pictorial layers from their reflectance spectra is explored. The use of a multi-analytical approach with the combination of Raman spectroscopy, laser-induced breakdown spectroscopy (LIBS), optical coherence tomography (OCT), FORS, and Vis-NIR multispectral reflectance imaging on single layers of ten pure acrylic colors revealed the existence of a clear correlation between the spectral response of diffuse reflectance and the micrometer thickness of acrylic paint layers.

In the paper by Aguilar-Rodríguez et al. [12], the combination of different analytical techniques (O.M., SEM-EDS, NMR spectroscopy, attenuated total reflectance (ATR) FTIR, micro-ATR-FTIR, and GC/MS) allowed for the characterization of the composition and painting technique of the mural Paisaje Abstracto painted in 1964 by Rafael Coronel with an unknown synthetic medium on a wood panel support. This study shows an innovative painting technique with poly(methyl methacrylate) (pMMA) as a binder and hypothesizes that a higher pMMA/MMA proportion could affect the mechanical properties and preservation of the mural, producing a rigid pictorial layer that fractures easily.

Studying paints and their components, such as binders, dyes, or pigments, may allow us to acquire useful information to differentiate original from non-original materials and date objects and artifacts. In the work of Macchia et al. [13], the combined use of noninvasive in situ techniques, such as portable O.M. and multispectral imaging, and nondestructive laboratory techniques, namely ATR-FTIR and SEM-EDS, allowed us to obtain information on the compositional nature of the paints of two vehicles in the Museum of Communication in Frankfurt (Germany), which were designed for the German postal and telecommunications service. The study was also helpful in identifying authentic materials to be preserved and areas that needed restoration to preserve the historical and testimonial importance of these vehicles.

The study and preservation of objects, infrastructures, and works belonging to the industrial archeological heritage is a relatively new area of research that emerged in the 1970s. Costantini et al. [14] aimed to identify the original color of the Ondarroa footbridge in Spain with a view to its future restoration. Raman spectroscopy and µ-EDXRF were used to characterize the pictorial layers of the footbridge. Colorimetric analyses were also performed to discover the different pigments used. The authors were also able to highlight the atmospheric impact on the preservation of the rotating bridge due to both the effect of marine aerosol and the presence of acidic compounds in the environment from anthropogenic activities.

Contemporary art is affected by a globally influenced, culturally diverse, and technologically advanced world in which it is increasingly easy to communicate and exchange information. Because of this, contemporary artworks are a dynamic combination of materials, methods, concepts, and subjects. The conservation of contemporary artworks has become a new and exciting field of study, which needs continuous research because of the ever-changing and more recent materials. The work of Alp et al. [15] provides preliminary knowledge about the materials of works by contemporary artist Paolo Gioli. It diagnoses Polaroid emulsion transfers via noninvasive analysis with FORS, Raman, and FTIR spectroscopies.

The work of Longoni et al. [16] confirms the importance of scientific investigations in responding to specific conservation and restoration problems of modern and contemporary artworks. The authors, after an initial visual inspection in visible and ultraviolet (U.V.) light, apply noninvasive and micro-invasive techniques (XRF spectroscopy, FTIR, Raman spectroscopy, and SEM-EDS) to investigate the materials in a silver monochromatic painting by Lucio Fontana, preserved in the exhibition hall of the San Fedele Church in Milan (Italy). The analytical data obtained made it possible to identify the composition of the metallic varnish and the underlying dark layer, both from the point of view of the pigments and binders used.

The review by Creydt and Fischer [17] discusses using omics technologies and various sampling techniques for analyzing written artifacts. The authors show that each omics strategy brings different information allowing better interpretation of data and a significant increase in knowledge of the written heritage.

Two reviews concern lipid residues and their degradation products, such as fatty acids and metal soaps, which are of great importance for the study of archeological objects and oil paintings [18,19]. Organic archeological residues can provide important information about past cultures, such as dietary habits, rituals, and medical practices [19]. In particular, Filopolou et al.’s [18] review explores the possibility of using infrared spectroscopy to investigate and distinguish fatty acids and their metal soaps. The authors show the diagnostic use of the spectroscopic characteristics of some typical fatty monoacids, diacids, and their Ca, Na, and Zn salts.

The review by Irto et al. [19] reports on artificial aging studies to elucidate the mechanisms of lipid degradation in archeological contexts and discusses methodologies for sampling and extracting specific lipid biomarkers in ancient ceramics. The authors stress the need to introduce innovative, miniaturized protocols to reduce the use of chemicals and avoid extractions with organic solvents, which are often laborious and environmentally unfriendly.
